# RETRACTION: PAK5 Facilitates the Proliferation, Invasion and Migration in Colorectal Cancer Cells

**DOI:** 10.1002/cam4.70520

**Published:** 2024-12-23

## Abstract

RETRACTION: S. Huang, Y. Zhu, C. Wang, X. Li, X. Cui, S. Tu, Lijuan You, J. Fu, Z. Chen, W. Hu and W. Gong, “PAK5 Facilitates the Proliferation, Invasion and Migration in Colorectal Cancer Cells,” *Cancer Medicine* 9, no. 13 (2020): 4777–4790, https://doi.org/10.1002/cam4.3084.

The above article, published online on 08 May 2020 in Wiley Online Library (wileyonlinelibrary.com), has been retracted by agreement between the authors; the journal Editor‐in‐Chief, Stephen Tait; and John Wiley & Sons Ltd. The retraction has been agreed due to an overlap between the Flag‐wtPAK5 and Flag‐wtPAK5 K478M invasion images presented in Figure 5H. The authors were unable to locate the raw data for the images and have requested the article to be retracted. The editors have lost confidence in the results and conclusions of this article.

RETRACTION: S. Huang, Y. Zhu, C. Wang, X. Li, X. Cui, S. Tu, Lijuan You, J. Fu, Z. Chen, W. Hu and W. Gong, “PAK5 Facilitates the Proliferation, Invasion and Migration in Colorectal Cancer Cells,” *Cancer Medicine* 9, no. 13 (2020): 4777–4790, https://doi.org/10.1002/cam4.3084.

The above article, published online on 08 May 2020 in Wiley Online Library (wileyonlinelibrary.com), has been retracted by agreement between the authors; the journal Editor‐in‐Chief, Stephen Tait; and John Wiley & Sons Ltd. The retraction has been agreed due to an overlap between the Flag‐wtPAK5 and Flag‐wtPAK5 K478M invasion images presented in Figure 5H. The authors were unable to locate the raw data for the images and have requested the article to be retracted. The editors have lost confidence in the results and conclusions of this article.

